# A GC-MS Protocol for the Identification of Polycyclic Aromatic Alkaloids from Annonaceae

**DOI:** 10.3390/molecules27238217

**Published:** 2022-11-25

**Authors:** Ilya A. P. Jourjine, Carolin Bauernschmidt, Christoph Müller, Franz Bracher

**Affiliations:** Department of Pharmacy—Center for Drug Research, Ludwig-Maximilians University Munich, Butenandtstraße 5-13, 81377 Munich, Germany

**Keywords:** alkaloids, Annonaceae, azafluorenones, diazafluoranthenes, oxoaporphines, azaoxoaporphines, gas chromatography, mass spectrometry

## Abstract

The Annonaceae are an old family of flowering plants belonging to the order Magnoliales, distributed mainly in tropical regions. Numerous Annonaceae species find ethnobotanical use for curing a broad range of diseases, among them cancer and infections by diverse pathogens. Hence, bioactive natural products from Annonaceae have received considerable interest in drug development. Beyond cytotoxic acetogenins, unique aporphine-derived polycyclic aromatic alkaloids are characteristic constituents of Annonaceae. Among them are unique tri- and tetracyclic aromatic alkaloids like azafluorenones, diazafluoranthenes, azaanthracenes, and azaoxoaporphines. The complex substitution pattern of these alkaloids represents a major challenge in structure elucidation of isolated natural products. Based on a broad spectrum of alkaloids available from our previous work, we present a GC-MS protocol for the identification of over 20 polycyclic aromatic alkaloids from Annonaceae. This collection of data will contribute to the future identification of the metabolite patterns of extracts from Annonaceae as an important source of novel bioactive secondary metabolites.

## 1. Introduction

The Annonaceae are an old family of flowering plants, mainly trees and shrubs, from the order Magnoliales. Found mainly in tropical regions, they have considerable economic significance as sources of edible fruits (custard apple, cherimoya, soursop) and essential oils (e.g., ylang-ylang oil from the flowers of *Cananga odorata* used in perfumes) among local populations. Furthermore, extracts (mainly from barks and roots) have found ethnobotanical use in the curing of a broad range of diseases, among them infections by diverse pathogens and tumors [1].

**Figure 1:**
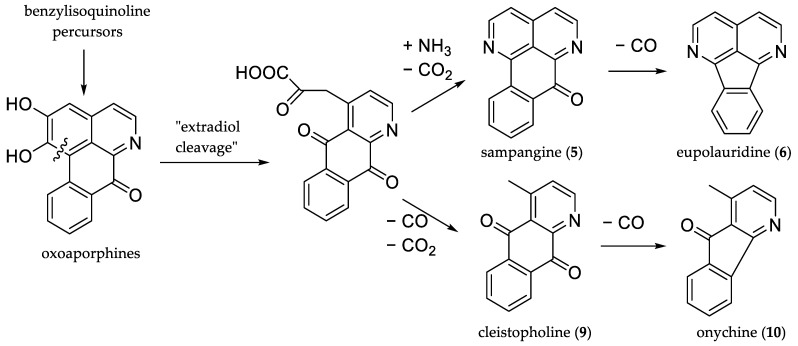
Postulated biosynthesis of azaoxoaporphines (e.g., sampangine, (**5**)), diazafluoranthenes (e.g., eupolauridine, (**6**)), azaanthraquinones (e.g., cleistopholine, (**9**)), and azafluorenones (e.g., onychine, (**10**)) starting from aporphinoid precursors.

Over decades, unique bioactive secondary metabolites have been isolated from Annonaceae. Acetogenins, a class of C_35_/C_37_ metabolites derived from long chain (C_32_/C_34_) fatty acids in the polyketide pathway, represent a very prominent chemotype among Annonaceae constituents. Exhibiting impressive antitumor activity, they, nonetheless, never reached the market due to severe toxicity, especially neurotoxicity [2]. A second prominent group of natural products from Annonaceae which received considerable interest in drug development are polycyclic aromatic alkaloids, most of which are biosynthetically derived from aporphinoid precursors. Oxoaporphines, well-known tetracyclic metabolites from the benzylisoquinoline pathway, are speculated [3,4] to undergo further transformations—including ring cleavage—during late-stage metabolism, as depicted in Figure 1. These proposed biotransformations produce unique tri- and tetracyclic aromatic alkaloids like azafluorenones, diazafluoranthenes, and azaoxoaporphines. Typical of the biosynthetically well-studied tyrosine-derived aporphinoid alkaloids, these natural products bear characteristic hydroxylation and methoxylation patterns.

In its infancy, structural analysis of natural products was primarily reliant on chemical degradation and derivatization reactions of the target molecules, analytical methods which have since been largely replaced by modern alternatives including multidimensional NMR (nuclear magnetic resonance), UV (ultraviolet), IR (infrared), CD (circular dichroism), X-ray crystallography, and MS (mass spectrometry) data, or proof of postulated structures by unambiguous total synthesis [5]. Naturally, structure elucidation is rarely based on a single analytical method but, rather, on mutually supportive data gathered from multiple sources. However, even despite these advancements, structural misassignments of alkaloids from this pathway occurred in the past [6,7,8,9], and identification of the precise substitution patterns of newly isolated Annonaceae alkaloids remains challenging. For example, the structures of annonaceous 4-aza-1-methylfluorenones onychine (**10**), first isolated from *Onychopetalum amazonicum*, and 6-methoxyonychine (**13**), first isolated from *Guatteria dielsiana*, were misreported as 1-aza-4-methylfluorenones on the basis of IR, UV, EIMS, HRMS, and ^1^H NMR data [10,11]. These structure misassignments were retained in the literature across several years in the 1980s until revision by total synthesis of the proposed structures [6]. We also showed that the postulated [11] and later revised [6] structure for 4-azafluorendione dielsine isolated from *Guatteria dielsiana* as 4-methyl-1*H*-indeno[1,2-*b*]pyridine-2,5-dione remains erroneous through total synthesis in previous works of ours [9]. Phytochemical analysis of newly investigated compounds from Annonaceae has to, therefore, rely on precise knowledge on previously isolated natural products with confirmed structures. Based on a representative library of synthesized alkaloids from decades of our own research in this field, we developed a convenient GC-MS (gas chromatography–mass spectrometry) protocol for the identification of polycyclic aromatic alkaloids from Annonaceae. Further, we included a number of synthetic congeners which have not (or not yet) been identified as natural products. In total, we present chromatographic and mass spectrometric data for 25 compounds of interest.

## 2. Results and Discussion

### 2.1. Source of Analytes

**Figure 2:**
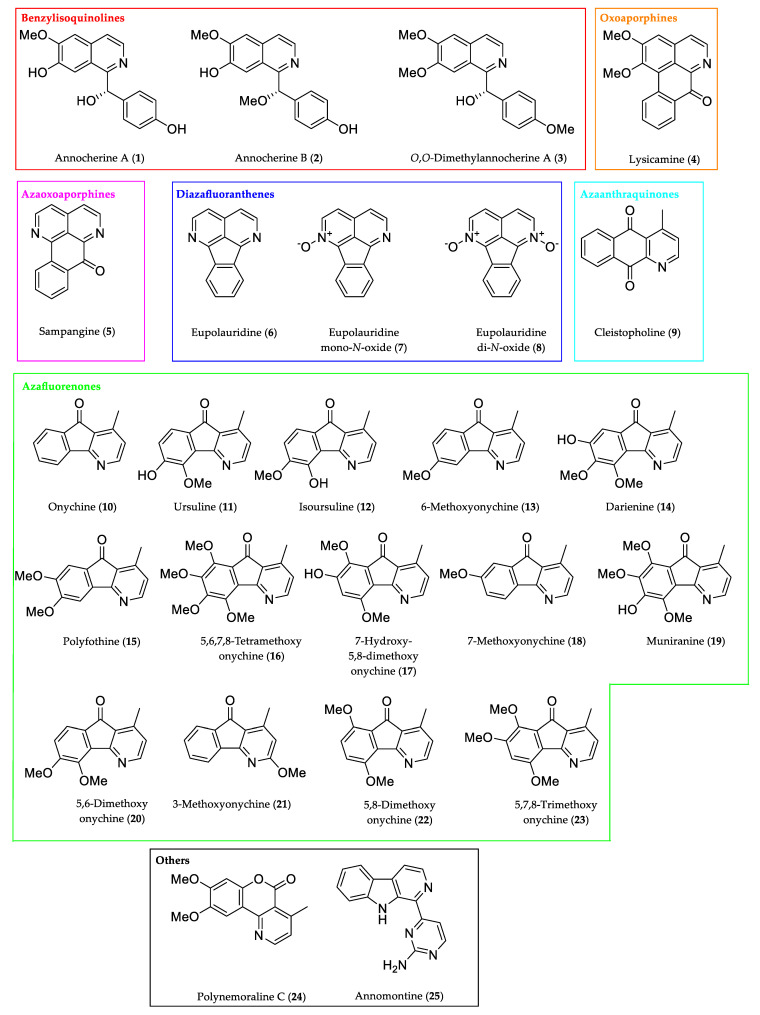
Structures of the alkaloids (and synthetic analogues) analyzed in this investigation.

Based on long-term work on the total synthesis of alkaloids from Annonaceae, we compiled an in-house substance library of 25 relevant compounds (Table 1 and Figure 2), covering all relevant chemotypes of aporphine-derived aromatic alkaloids typical of (some of them even unique to) Annonaceae. It includes representatives of well-known, broadly distributed alkaloid classes (1-benzylisoquinolines, oxoaporphines) as well as aporphine-derived chemotypes (azaoxoaporphine, diazafluoranthene, azaanthraquinone, azafluorenone) characteristic of Annonaceae. Our efforts also furnished the unique lactone alkaloid polynemoraline C (**24**). Interestingly, this alkaloid was found to be accompanied by several aporphinoid alkaloids in *Polyalthia nemoralis* (Annonaceae) [12]. With the pyrimidyl-β-carboline annomontine (**25**) [13] we also included an Annonaceae alkaloid which arises from a distinct biosynthetic pathway (tryptophan-derived). Representative citations for the biological sources of these alkaloids are given in Table 1; information about the total syntheses is given in Section 3.1 Materials and Reagents.

The alkaloid collection was supplemented by a couple of synthetic azafluorenones which have not yet been identified as natural products. (Note: 5,6-Dimethoxyonychine (**20**) and 5,8-dimethoxyonychine (**22**) were mentioned as “alkaloids” by Zhang [7]. In fact, these compounds were only synthesized in order to compare the spectroscopic data with those of alkaloids isolated from *Oxandra xylopioides*.)

### 2.2. GC-MS Analysis

A 5% phenyl polymethylsiloxane fused-silica capillary column was used as the stationary phase due to its well-known high separation efficiency, robustness, and its broad use in GC-MS analysis. To determine the retention behavior of each substance, we used the RRT in relation to the internal standard (IS) fluorene and the temperature-programmed Kováts retention index (or linear retention index, LRI) (Equation (1)).

(1) $$I=100\times \left(\frac{t_{Ri}-t_{Rz}}{t_{R\left(z+1\right)}-t_{Rz}}+z\right)$$

I = Kováts retention index
$t_{Ri}$ = total retention time of the substance
$t_{Rz}$ = total retention time of the n-alkane
z = number of the carbon atoms of the n-alkane
$t_{R\left(z+1\right)}$ = total retention time of the n-alkane + 1 carbon atom
**Equation 1.** Determination of Kováts retention indices in case of temperature-programmed gas chromatography according to IUPAC (International Union of Pure and Applied Chemistry) [27].

As a proof of concept, the calculated indices of fluorene (**IS**), lysicamine (**4**), and onychine (**10**) were compared to published indices in the NIST (National Institute of Standards and Technology) Database [28]. We determined for **IS** a LRI of 1599 iu. The NIST Database estimated a retention index of 1494 iu and determined an average index of 1574 iu for 5% phenyl polymethylsiloxane capillary columns, which is close to our determined index. For lysicamine (**4**) and onychine (**10**), the NIST Database only estimated Kováts retention indices of 2465 iu and 1772 iu; our experimentally determined linear retention indices were 3258 iu and 1823 iu. For all other substances, no Kováts retention indices were available in the NIST Database. The linear retention indices of 25 polycyclic aromatic alkaloids from Annonaceae and related compounds are provided in Table 1. Additional chromatographic parameters of the analytes are listed in Table S1 (Supplementary Material).

**Figure 3:**
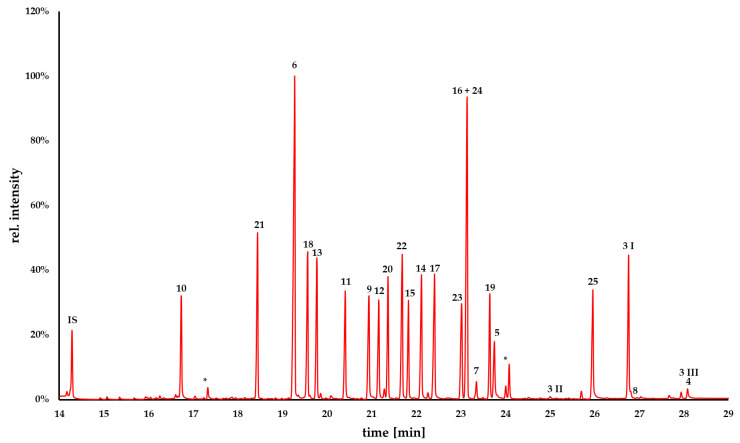
Total ion chromatogram of the compounds **3**–**25**; **3** *O*,*O*-dimethylannocherine A, **4** lysicamine, **5** sampangine, **6** eupolauridine, **7** eupolauridine mono-*N*-oxide, **8** eupolauridine di-*N*-oxide, **9** cleistopholine, **10** onychine, **11** ursuline, **12** isoursuline, **13** 6-methoxyonychine, **14** darienine, **15** polyfothine, **16** 5,6,7,8-tetramethoxyonychine, **17** 7-hydroxy-5,8-dimethoxyonychine, **18** 7-methoxyonychine, **19** muniranine, **20** 5,6-dimethoxyonychine, **21** 3-methoxyonychine, **22** 5,8-dimethoxyonychine, **23** 5,7,8-trimethoxyonychine, **24** polynemoraline C, **25** annomontine; * impurities. Due to the individual volatility and thermal stability of the compounds, different concentrations were used in order to obtain a chromatogram visualizing the chromatographic behavior of all analytes (**3** 100 µg/mL, **4–6** 20 µg/mL, **7**, **8** 50 µg/mL, **9–25** and **IS** 20 µg/mL.

Using a moderate heating rate (10 °C/min), it was possible to separate nearly all analytes of interest. The separation of the alkaloids 5,6,7,8-tetramethoxyonychine (**16**) and polynemoraline C (**24**) (Figure 2) was impossible based on their retention behavior. Both substances showed a similar RRT of 1.621 and 1.617 and Kováts retention indices 2536 iu and 2528 iu, respectively (Figure 3). However, they differed significantly in their mass spectra, which made identification easy (base peaks *m*/*z* 315 and *m*/*z* 271, respectively). The use of stationary phases with a modified selectivity (e.g., mid-polar columns with 50% phenyl 50% methylpolysiloxane) might allow separation of co-eluting compounds like (**16**) and (**24**).

Similarly, the differentiation of the benzylisoquinolines annocherine A (**1**) and annocherine B (**2**) based on their respective retention behavior and their mass spectra failed (see Supplementary Material). Due to their poor volatility, the peak intensities were low, even at an inlet temperature of 300 °C. The substances were not detectable at lower inlet temperatures, and the use of 300 °C inlet temperature led to degradation into **1I**/**1II** and **2I**/**2II** during injection (see chromatograms in the Supplementary Material). Fragments **1I**/**2I** showed the same RRT of 1.818, and a base peak at *m*/*z* 280. Even **1II**/**2II** showed the same RRT of 1.985 and a base peak at *m*/*z* 279. Differentiation on the basis of the mass spectra also failed. Both substances showed similar MS spectra (EI, 70 eV). The molecular ion peak [M^+^] of **1** (*m*/*z* 279) and **2**) (*m*/*z* 311) could not be observed in any mass spectrum of **1I**/**1II** and **2I**/**2II**. The volatility of the third benzylisoquinoline, *O*,*O*-dimethylannocherine A (**3**) was higher compared to **1** and **2**. However, alkaloid **3** partially degraded into **3II** and **3III** during injection and the [M^+^] at *m*/*z* 325 could not be observed. Thus, the investigated 1-benzylisoquinolines proved challenging for GC-MS analysis. The most likely cause is the presence of the uncommon hydroxy/methoxy substituent at the benzylic sp^3^ carbon, which leads to fragmentation under the thermal stress conditions in the injector block of the gas chromatograph. We suppose that the driving force for these thermal fragmentations is the formation of highly conjugated systems like enamines and *p*-quinone methides (Figure S1, Supplementary Material). Since *p*-quinone methide formation is not plausible with the *p*-methoxybenzyl compound **3**, this alkaloid exhibits higher thermal stability compared to the *p*-hydroxybenzyl alkaloids **1** and **2**.

Fortunately, eupolauridine mono-*N*-oxide (**7**) and eupolauridine di-*N*-oxide (**8**) were degraded in a plausible manner during injection. The chromatogram of the mono-*N*-oxide (**7**) showed a peak of the mono-*N*-oxide (15%) and a peak of deoxygenation product eupolauridine (**6**, 85%), whereas eupolauridine di-*N*-oxide (**8**, 5%) was degraded into the mono-*N*-oxide (**7**, 30%) and eupolauridine (**6**, 65%; see chromatograms in the Supplementary Material). The base peaks of both N-oxides were *m*/*z* 204, but [M^+^] peaks were present as well for both eupolauridine mono-*N*-oxide (**7**, *m*/*z* 220) and eupolauridine di-*N*-oxide (**8**, *m*/*z* 236). The differentiation of these alkaloids is possible, but thorough examination of the chromatograms is required to avoid misidentification of the degradation product as the analyte.

In summary, polycyclic aromatic alkaloids from Annonaceae were clearly identifiable except for the aforementioned benzylisoquinolines (**1**–**3**). Out of 25 substances, 14 showed a molecular ion peak [M^+^] identical to the base peak (**4**–**6**, **9**–**11**, **13**, **15**–**18**, **23**–**25**), which facilitates identification.

## 3. Materials and Methods

### 3.1. Materials and Reagents

The analytes (see Table 1 and Figure 2) were taken from our in-house substance library, compiled from previous and very recent work on natural product total synthesis, including a novel radical cyclization approach for azafluorenones [29]. All other syntheses are described in detail in our previous publications:

Azafluorenone alkaloids onychine (**10**) [30], ursuline (**11**) [29], isoursuline (=oxylopine; **12**) [29], 6-methoxyonychine (**13**) [31,32], darienine (**14**) [29], polyfothine (**15**) [29], 5,6,7,8-tetramethoxyonychine (**16**) [29], 7-hydroxy-5,8-dimethoxyonychine (**17**) [29], 7-methoxyonychine (**18**) [29], and muniranine (**19**) [29], as well as synthetic analogues 5,6-dimethoxyonychine (**20**) [29], 3-methoxyonychine (**21**) [9], 5,8-dimethoxyonychine (**22**) [29], and 5,7,8-trimethoxyonychine (**23**) [29].

Sampangine (**5**) [33,34] is the most prominent member of azaoxoaporphine alkaloids, eupolauridine (**6**) [30] as well as its mono- (**7**) and di-*N*-oxide (**8**) [35] are representatives of the diazafluoranthene alkaloids, and cleistopholine (**9**) [33] was the first azaanthraquinone alkaloid isolated from Annonaceae. As representatives of the postulated biosynthetic precursors of these unique aromatic alkaloids, we included the oxoaporphine lysicamine (**4**) [36] and three 1-benzylisoquinoline alkaloids: annocherine A (**1**), annocherine B (**2**), and *O*,*O*-dimethylannocherine A (=annocherine D; **3**) [36], the latter of which is an alkaloid that, to date, has only been isolated from Nelumbonaceae plants.

Further, we included the annonaceous lactone alkaloid polynemoraline (**24**) [29] and the pyrimidyl-β-carboline alkaloid annomontine (**25**) [37,38] in the investigation.

For the determination of the temperature-programmed Kováts retention indices, alkane standards C7–C30 saturated (1000 mg/mL in *n*-hexane), alkane standard C10–C40 (all even, 50 mg/mL in *n*-heptane), as well as acetonitrile of HPLC (high performance liquid chromatography)-grade were obtained from Sigma Aldrich (Schnelldorf, Germany). The internal standard (**IS**) fluorene was purchased from HPC Standards GmbH (Cunnersdorf, Germany).

Stock solutions of each compound (1 mg/mL) were prepared in acetonitrile and stored at 4 °C. Working solutions (in acetonitrile) for each experiment were prepared in following concentrations: compounds **4**–**6** and **9**–**25**, and **IS** at 20 µg/mL; compounds **7** and **8** at 50 µg/mL; compounds **1**–**3** 100 µg/mL.

### 3.2. Instruments and Equipment

Gas chromatography (GC) was performed with a 7820A gas chromatograph from Agilent Technologies (Santa Clara, CA, USA). The G4514A autosampler and the G4513A injector were from Agilent Technologies (Santa Clara, CA, USA). Instrument control and data analysis were carried out with a Masshunter 8.0 from Agilent. An HP-5-ms capillary column of 30 m length, 0.25 mm i.d., and 0.25 µm film thickness was used at a constant flow rate of 1.2 mL/min. Helium 99.999% from Air Liquide (Düsseldorf, Germany) was used as carrier gas. The inlet temperature was kept at 300 °C and the injection volume was 2 µL with splitless time 0.5 min. The initial column temperature was 50 °C and held for 1 min. Then, temperature was ramped up to 320 °C (10 °C/min) and held for 2 min. The total run time was 30 min. MS source temperature was 230 °C and quadrupole temperature was 150 °C. The MS was operated with electron ionization (EI) at 70 eV in scan mode (*m*/*z* 40–600) with a solvent delay of 7.0 min.

## 4. Conclusions

The herein described gas-chromatographic system is able to separate annonaceous alkaloids from all relevant chemotypes (see Figure 1) efficiently, and in combination with MS detection represents a powerful and fast tool for the unambiguous identification of known alkaloids. By collecting chromatographic and mass spectroscopic data for an unprecedentedly large library on annonaceous alkaloids, we present here a valuable database for other scientists in this field. This method is only limited by the thermal instability of single chemotypes (benzylisoquinolines with benzylic oxygenation, *N*-oxides), where a very careful analysis of chromatographic and MS data is afforded. Since reports on the identification of novel alkaloids from Annonaceae continue to be published, our work should further contribute to the fast and secure analysis of alkaloid patterns and alleviate the structure elucidation of hitherto unknown alkaloids. Furthermore, the inclusion of some synthetic analogues (especially azafluorenones) which have not (yet) been identified as natural products might contribute to structure elucidations pertaining to the concept of “natural product anticipation through synthesis” [39].

## Figures and Tables

**Table 1:** Alkaloids (and synthetic analogues) analyzed in this investigation, including information on biological sources, chemical structure, MS data, and chromatographic properties (see Figure 3). * Not described as a natural product; ** not isolated from Annonaceae, but from Nelumbonaceae; ^1^ CAS = Chemical Abstract Services; ^2^ RTT = relative retention time; ^3^ temperature-programmed Kováts retention index.

Code	Trivial Name [Biological Source]	CAS ^1^ Number	Chemical Formula	M [g/mol]	Characteristic Ions [**m*/*z**] (Base Peak in Bold)	RRT ^2^ (Fluorene)	Kováts Index ^3^
**1**	Annocherine A [14]	344928-12-7	C_17_H_15_NO_4_	297.10	(**I**) **280**, 265, 220	1.818	2925
					(**II**) **279**, 264, 236	1.985	3295
**2**	Annocherine B [14]	344928-13-8	C_18_H_17_NO_4_	311.12	(**I**) **280**, 265, 220	1.818	2925
					(**II**) **279**, 264, 236	1.985	3295
**3**	*O*,*O*-Dimethylannocherine A ** [15]	1268489-61-7	C_19_H_19_NO_4_	325.13	(**I**) 322, 308, **292**	1.872	3045
					(**II**) **308**, 294, 278	1.750	2784
					(**III**) 307, **292**, 248	1.937	3190
**4**	Lysicamine [16]	15444-20-9	C_18_H_13_NO_3_	291.09	**291**, 248, 177	1.964	3258
**5**	Sampangine [17]	116664-93-8	C_15_H_8_N_2_O	232.06	**232**, 204, 151	1.663	2614
**6**	Eupolauridine [18]	58786-39-3	C_14_H_8_N_2_	204.06	**204**, 177, 150	1.346	2074
**7**	Eupolauridine mono-*N*-oxide [19]	96889-95-1	C_14_H_8_N_2_O	220.06	220, **204**, 165	1.630	2552
**8**	Eupolauridine di-*N*-oxide [19]	96889-96-2	C_14_H_8_N_2_O_2_	236.06	236, 220, **204**	1.885	3072
**9**	Cleistopholine [19]	96889-94-0	C_14_H_9_NO_2_	223.06	**223**, 195, 167	1.467	2267
**10**	Onychine [10]	58787-04-5	C_13_H_9_NO	195.07	**195**, 166, 139	1.171	1823
**11**	Ursuline [20]	111316-34-8	C_14_H_11_NO_3_	241.07	**241**, 223, 183	1.430	2204
**12**	Isoursuline [21]	112368-57-7	C_14_H_11_NO_3_	241.07	241, 212, 198	1.481	2290
**13**	6-Methoxyonychine [11]	105418-67-5	C_14_H_11_NO_2_	225.08	**225**, 182, 154	1.386	2135
**14**	Darienine [20]	111316-27-9	C_15_H_13_NO_4_	271.08	271, **256**, 225	1.546	2400
**15**	Polyfothine [22]	122908-91-2	C_15_H_13_NO_3_	255.09	**255**, 212, 169	1.529	2371
**16**	5,6,7,8-Tetramethoxyonychine [23]	N/A	C_17_H_17_NO_5_	315.11	**315**, 300, 239	1.621	2536
**17**	7-Hydroxy-5,8-dimethoxyonychine [24]	N/A	C_15_H_13_NO_4_	271.08	**271**, 242, 172	1.566	2436
**18**	7-Methoxyonychine [25]	117719-70-7	C_14_H_11_NO_2_	225.08	**255**, 210, 154	1.369	2108
**19**	Muniranine [26]	N/A	C_16_H_15_NO_5_	301.10	301, 283, **200**	1.653	2596
**20**	5,6-Dimethoxyonychine *	112368-58-8	C_15_H_13_NO_3_	255.09	255, 254, **226**	1.494	2312
**21**	3-Methoxyonychine *	145013-64-5	C_14_H_11_NO_2_	225.08	225, **224**, 196	1.290	1989
**22**	5,8-Dimethoxyonychine *	112368-60-2	C_15_H_13_NO_3_	255.09	255, **254**, 226	1.516	2349
**23**	5,7,8-Trimethoxyonychine *	N/A	C_16_H_15_NO_4_	285.10	**285**, 270, 256	1.612	2519
**24**	Polynemoraline C [12]	1129491-73-1	C_15_H_13_NO_4_	271.09	**271**, 228, 185	1.617	2528
**25**	Annomontine [13]	82504-00-5	C_15_H_11_N_5_	261.10	**261**, 245, 220	1.816	2920

## Data Availability

Not applicable.

## Supplementary Materials

The following supporting information can be downloaded at https://www.mdpi.com/article/10.3390/molecules27238217/s1, Figure S1: Degradation; Table S1: Additional chromatographic parameters of the analytes; File S1: Chromatograms of the problematic compounds **1**, **2**, **3**, **7**, and **8**; File S2: Substance data sheets for the single analytes (including retention data and MS spectra), and a scheme showing proposed fragmentation patterns of 1-benzylisoquinoline alkaloids **1**–**3**.
