# Supplementary material for: A GC-MS Protocol for the Identification of Polycyclic Aromatic Alkaloids from Annonaceae
Source: Molecules. 2022 Nov 25;27(23):8217. doi: 10.3390/molecules27238217 (PMC9738936; doi:10.3390/molecules27238217)

Supplementary Material: **Chromatograms of compounds 1-3, 7, 8**

Annocherine A (1)

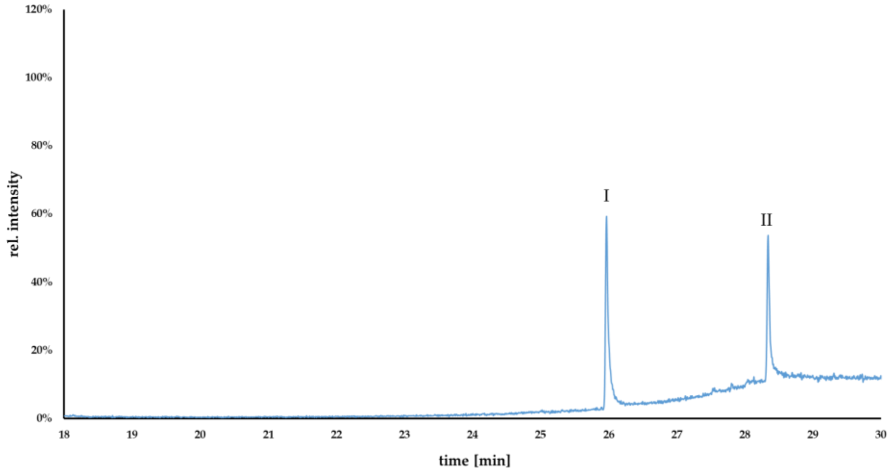

Annocherine B (2)

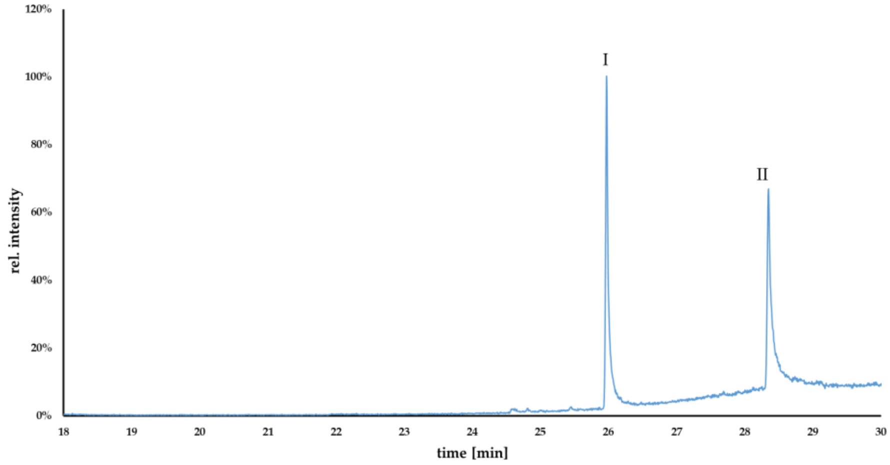

***O,O*- Dimethylannocherine A (3)**

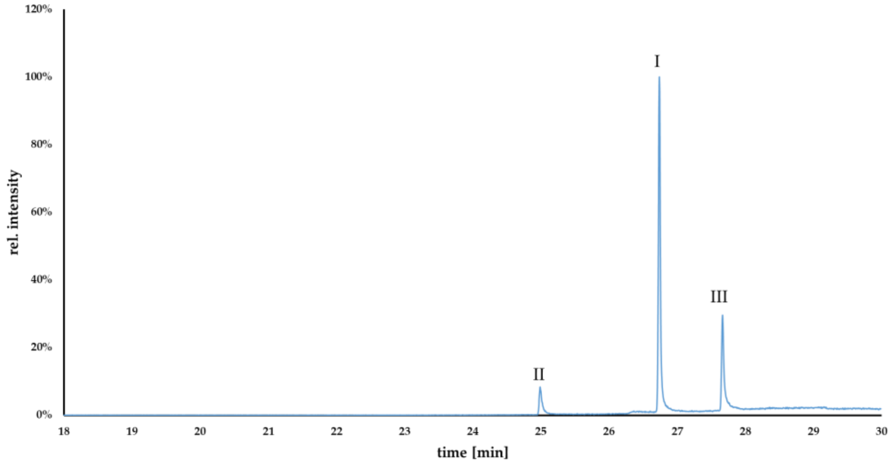

Eupolauridine mono-*N*-oxide (7)

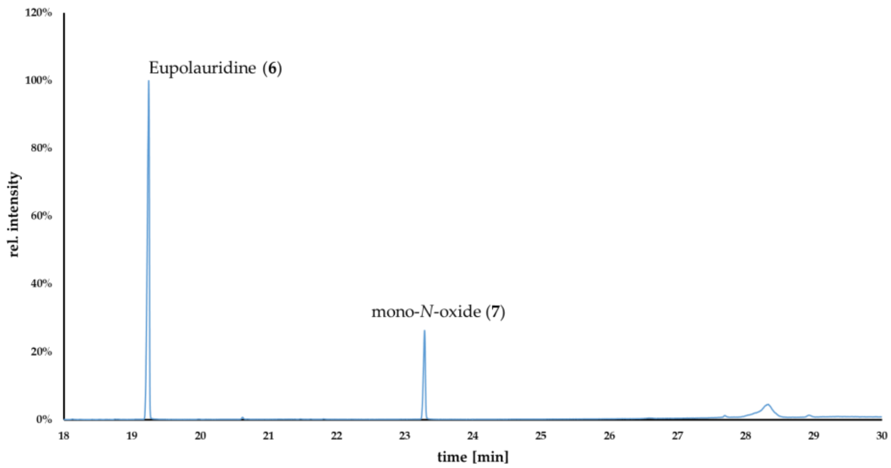

Eupolauridine di-*N*-oxide (8)

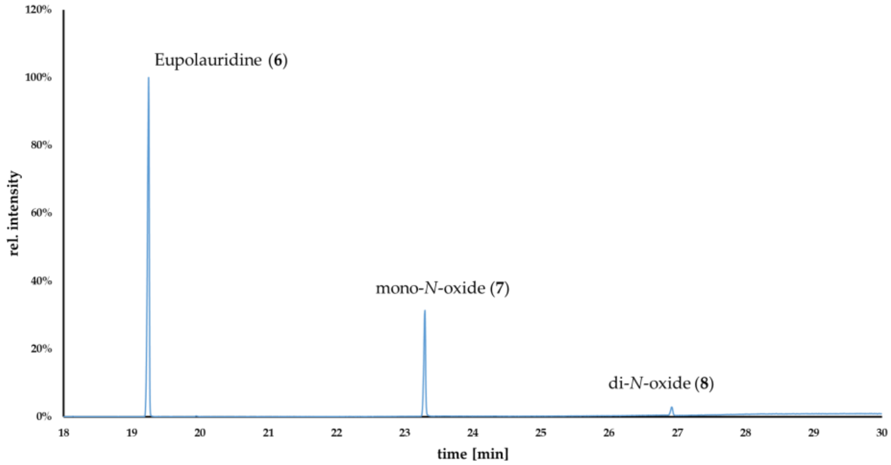

Supplement: Supplementary file 1 [file molecules-27-08217-s001.zip › Supplementary Material File S1. Chromatograms of compounds 1_2_3_7_8.pdf]
