# Supplementary material for: A GC-MS Protocol for the Identification of Polycyclic Aromatic Alkaloids from Annonaceae
Source: Molecules. 2022 Nov 25;27(23):8217. doi: 10.3390/molecules27238217 (PMC9738936; doi:10.3390/molecules27238217)

Supplementary Material: Substance data sheets

**Compound No.**	**Internal Standard (IS)**		
**Trival name**	**Fluorene**		
**CAS Number**	86-73-7	**M [g/mol]**	166.08
**Structure**	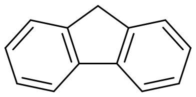	**Chemical formula**	$C_{13}H_{10}$
		**Kováts index [iu]**	1599
		**RRT (fluorene)**	1.000

Mass spectrum

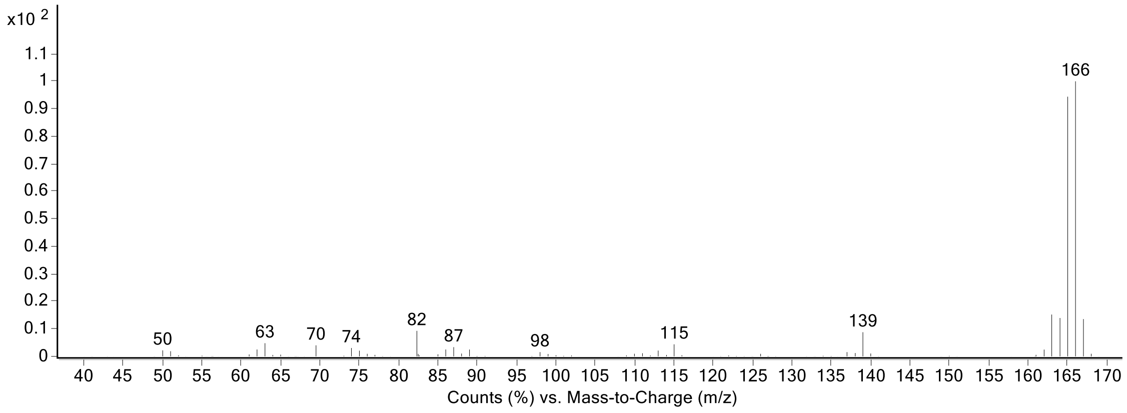

Compound No.			1
Trival name	Annocherine A		
CAS Number	344928-12-7	M [g/mol]	297.10
Structure	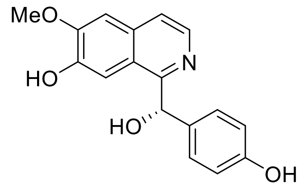	Chemical formula	$C_{17}H_{15}NO_4$
		Kováts index [iu]	(**I**) 2925<br>(**II**) 3295
		RRT (fluorene)	(**I**) 1.818<br>(**II**) 1.985

Mass spectrum

(I)

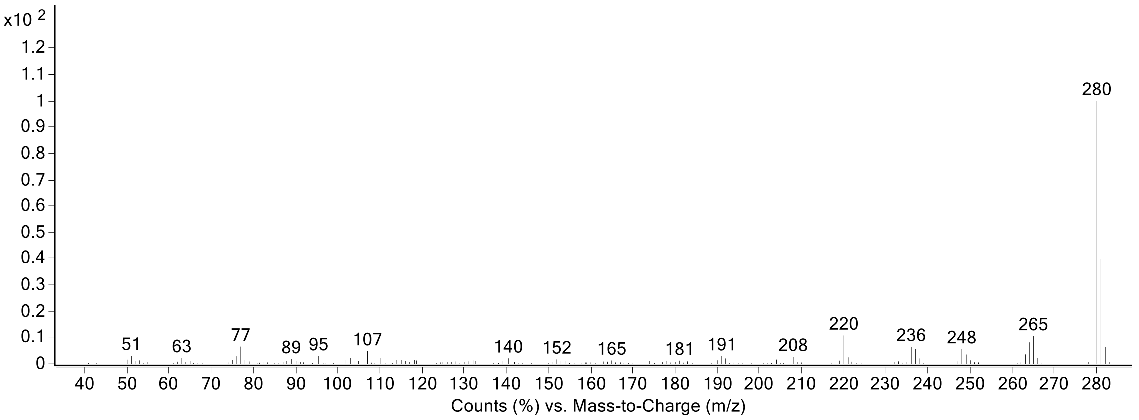

(II)

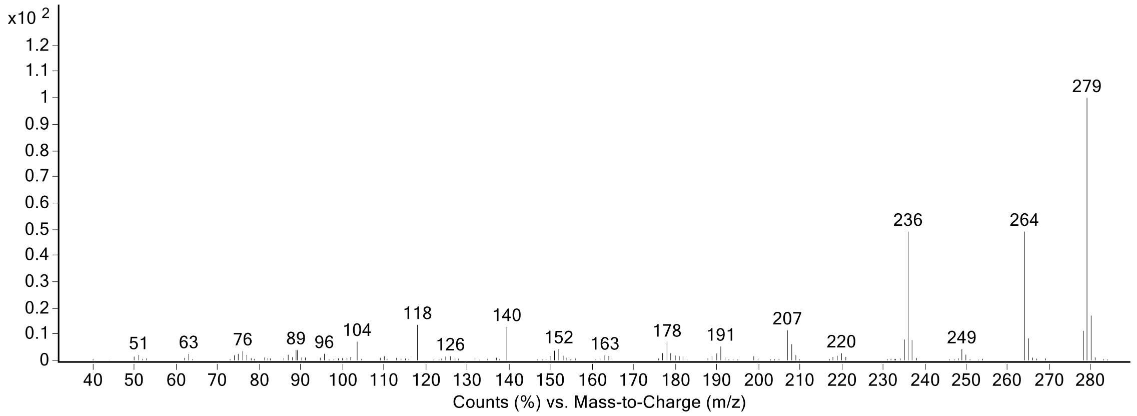

**Compound No.**	**2**		
**Trival name**	**Annocherine B**		
**CAS Number**	344928-13-8	**M [g/mol]**	311.12
**Structure**	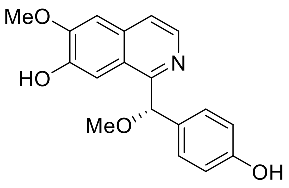	**Chemical formula**	$C_{18}H_{17}NO_4$
		**Kováts index [iu]**	(**I**) 2925<br>(**II**) 3295
		**RRT (fluorene)**	(**I**) 1.818<br>(**II**) 1.985

Mass spectrum

(I)

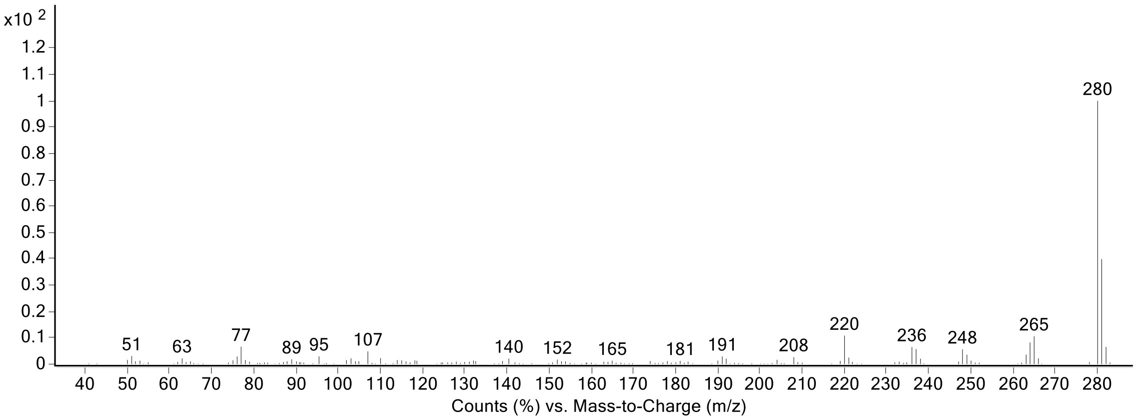

(II)

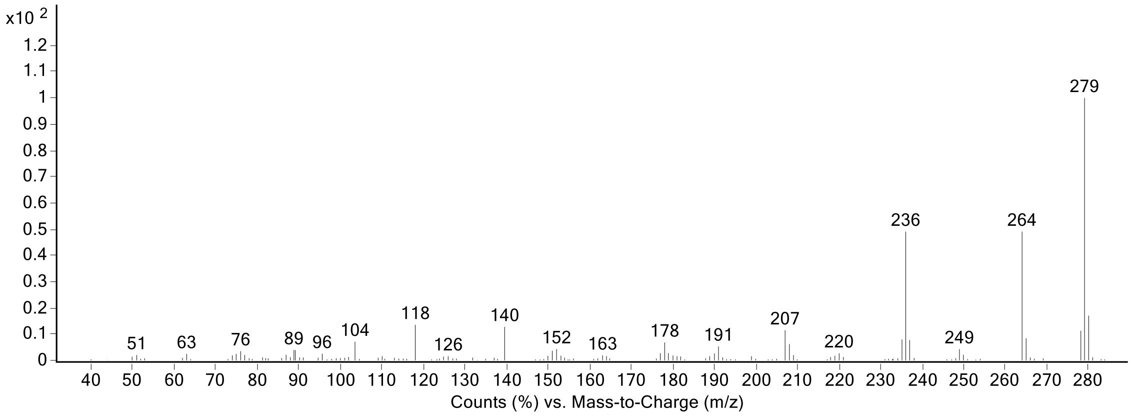

**Compound No.**	**3**		
**Trival name**	***O,O*-Dimethylannocherine A** (*syn.:* Annocherine D)		
**CAS Number**	1268489-61-7	**M [g/mol]**	325.13
**Structure**	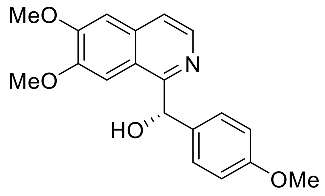	**Chemical formula**	$C_{19}H_{19}NO_4$
		**Kováts index [iu]**	(**I**): 3045<br>(**II**): 2784<br>(**III**): 3190
		**RRT (fluorene)**	(**I**): 1.872<br>(**II**): 1.750<br>(**III**): 1.937

Mass spectrum

(**I**)

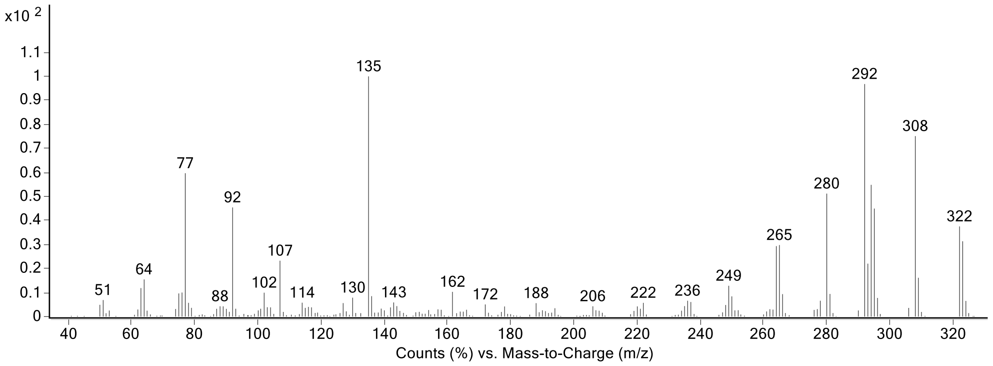

(**II**)

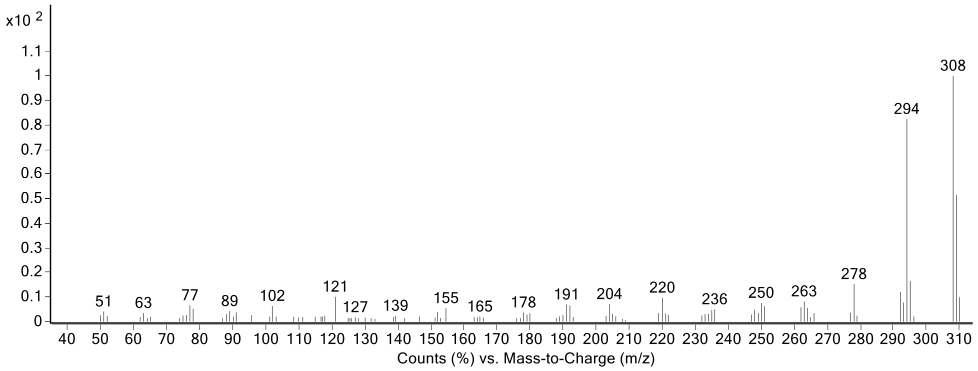

(**III**)

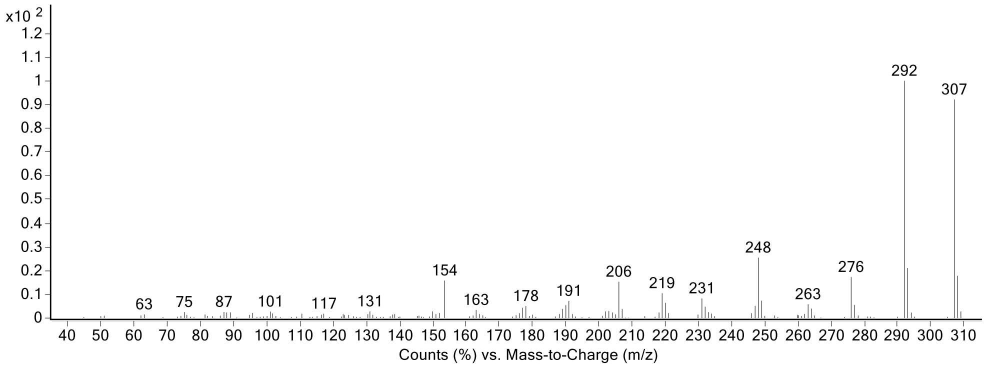

**Compound No.**	**4**		
**Trival name**	**Lysicamine**		
**CAS Number**	15444-20-9	**M [g/mol]**	291.09
**Structure**	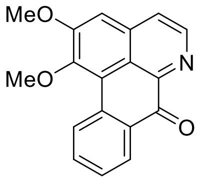	**Chemical formula**	$C_{18}H_{13}NO_3$
		**Kováts index [iu]**	3258
		**RRT (fluorene)**	1.964

Mass spectrum

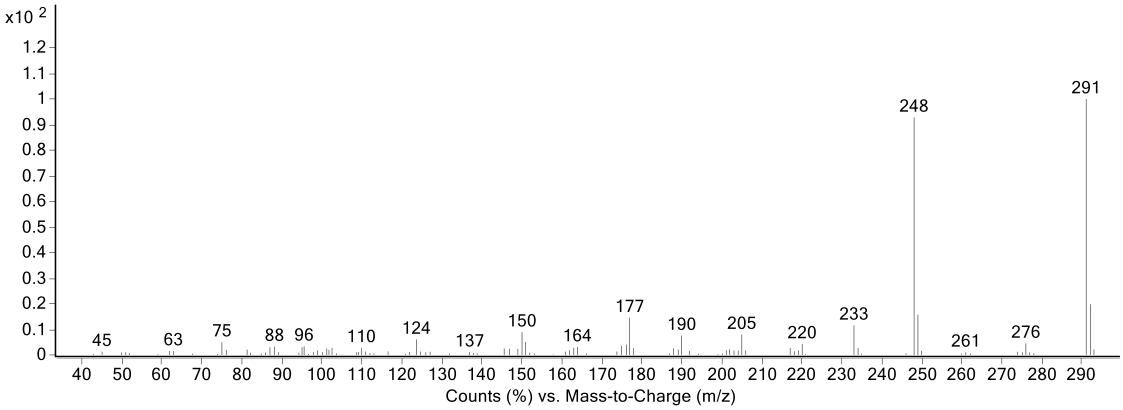

Compound No.	5		
Trival name	Sampangine		
CAS Number	116664-93-8	M [g/mol]	232.06
Structure	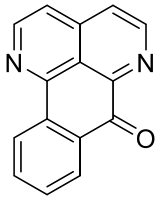	Chemical formula	$C_{15}H_8N_2O$
		Kováts index [iu]	2614
		RRT (fluorene)	1.663

Mass spectrum

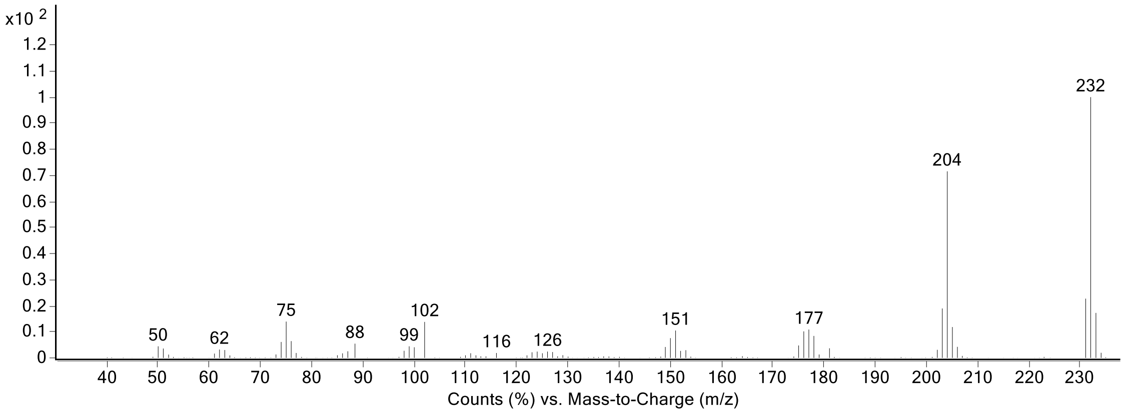

**Compound No.**			**6**
**Trival name**	**Eupolauridine**		
**CAS Number**	58786-39-3	**M [g/mol]**	204.06
**Structure**	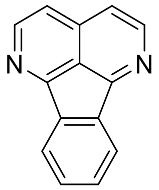	**Chemical formula**	$C_{14}H_8N_2$
		**Kováts index [iu]**	2074
		**RRT (fluorene)**	1.346

Mass spectrum

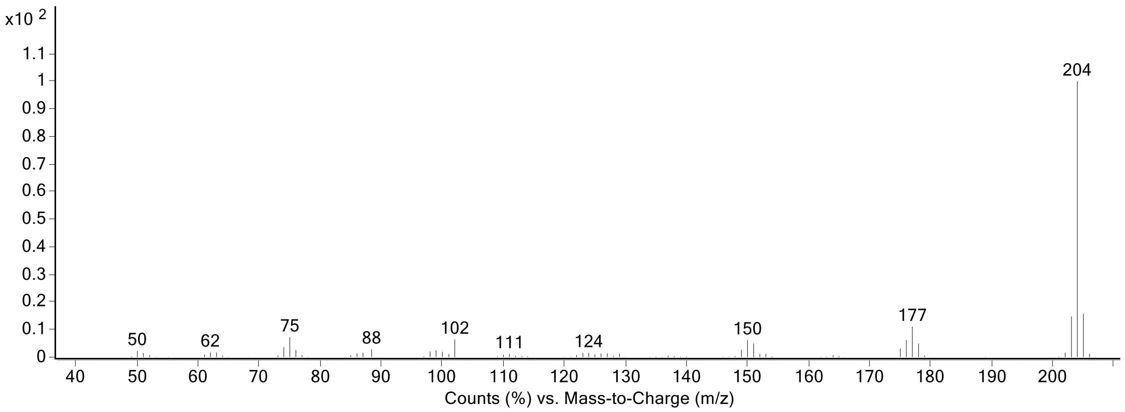

Compound No.			7
Trival name	Eupolauridine mono-*N*-oxide		
CAS Number	96889-95-1	M [g/mol]	220.06
Structure	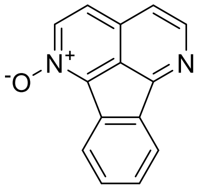	Chemical formula	$C_{14}H_8N_2O$
		Kováts index [iu]	2552
		RRT (fluorene)	1.630

Mass spectrum

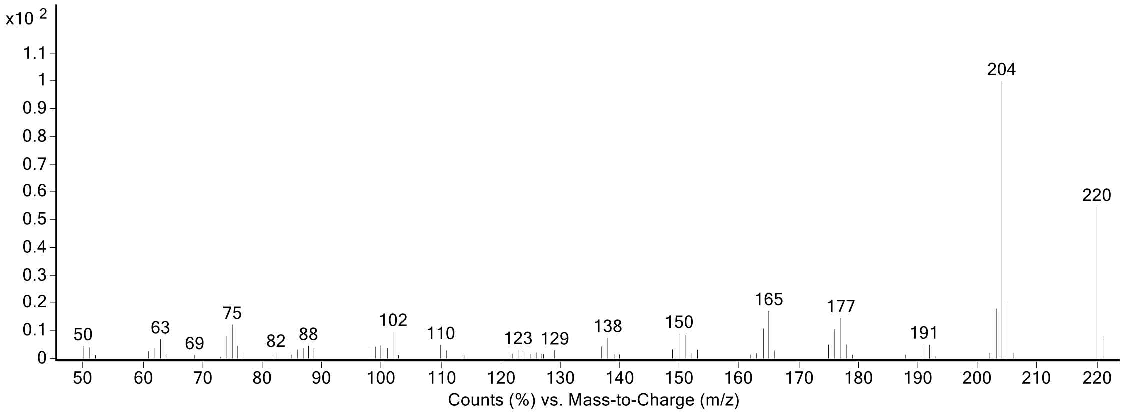

**Compound No.**	**8**		
**Trival name**	**Eupolauridine di-*N*-oxide**		
**CAS Number**	96889-96-2	**M [g/mol]**	236.06
**Structure**	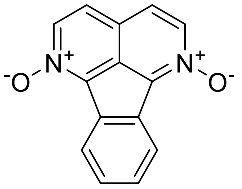	**Chemical formula**	$C_{14}H_8N_2O_2$
		**Kováts index [iu]**	3072
		**RRT (fluorene)**	1.885

Mass spectrum

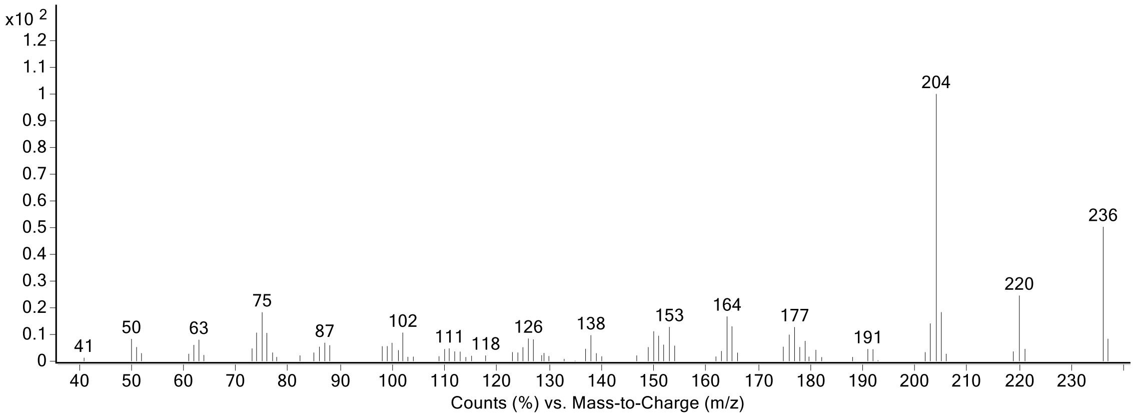

**Compound No.**	**9**		
**Trival name**	**Cleistopholine**		
**CAS Number**	96889-94-0	**M [g/mol]**	223.06
**Structure**	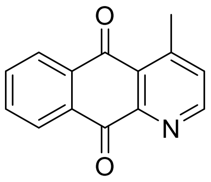	**Chemical formula**	$C_{14}H_9NO_2$
		**Kováts index [iu]**	2267
		**RRT (fluorene)**	1.467

Mass spectrum

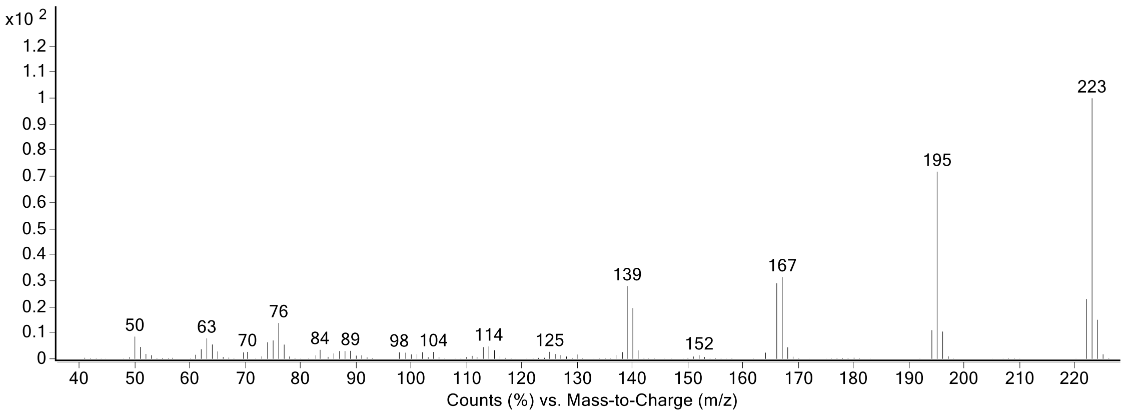

**Compound No.**	**10**		
**Trival name**	**Onychine**		
**CAS Number**	58787-04-5	**M [g/mol]**	195.07
**Structure**	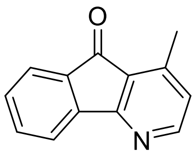	**Chemical formula**	$C_{13}H_{9}NO$
		**Kováts index [iu]**	1823
		**RRT (fluorene)**	1.171

Mass spectrum

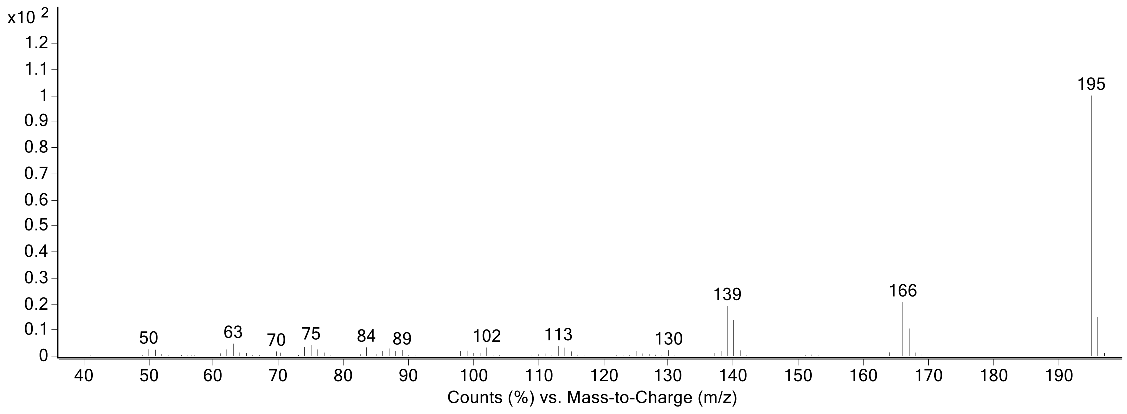

Compound No.	11
Trival name	Ursuline

CAS Number	111316-34-8	M [g/mol]	241.07
Structure	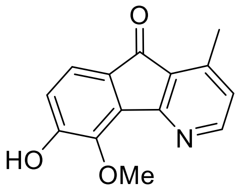	Chemical formula	$C_{14}H_{11}NO_3$
		Kováts index [iu]	2204
		RRT (fluorene)	1.430

Mass spectrum

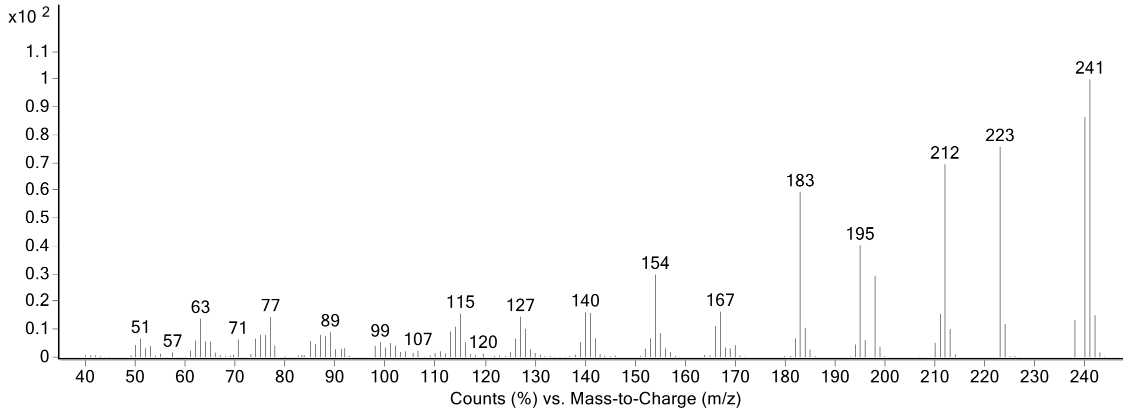

**Compound No.**	**12**		
**Trival name**	**Isoursoline** (*syn.:* Oxylopine)		
**CAS Number**	112368-57-7	**M [g/mol]**	241.07
**Structure**	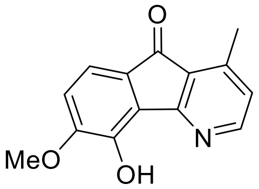	**Chemical formula**	$C_{14}H_{11}NO_3$
		**Kováts index [iu]**	2290
		**RRT (fluorene)**	1.481

Mass spectrum

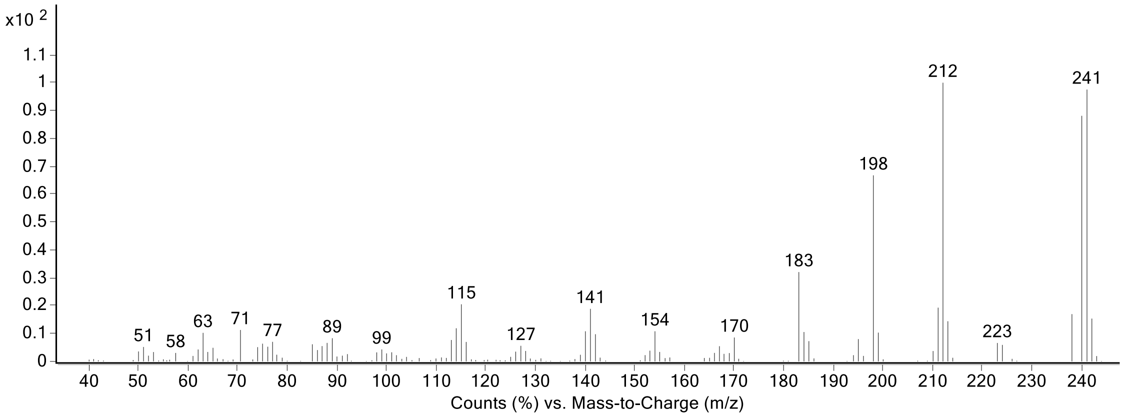

Compound No.	13		
Trival name	6-Methoxyonychine		
CAS Number	105418-67-5	M [g/mol]	225.08
Structure	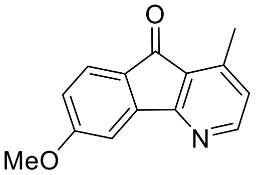	Chemical formula	$C_{14}H_{11}NO_2$
		Kováts index [iu]	2135
		RRT (fluorene)	1.386

Mass spectrum

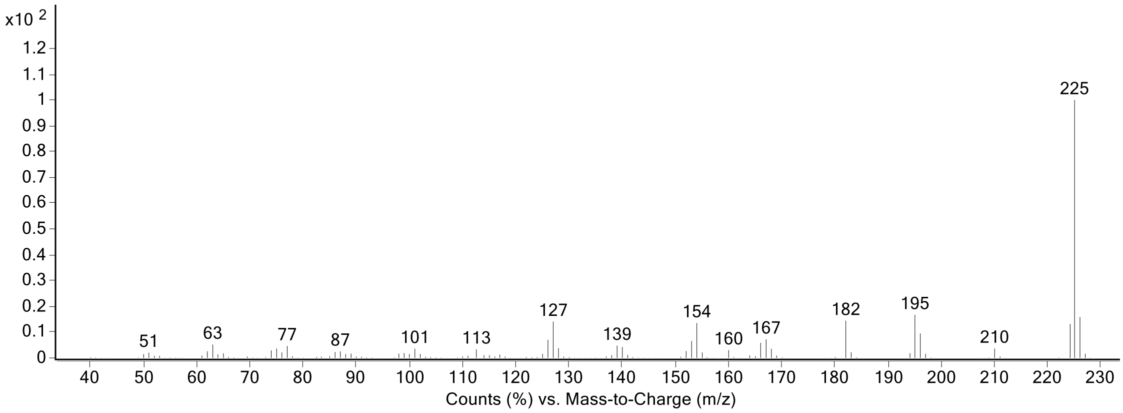

Compound No.			14
Trival name	Darienine		
CAS Number	111316-27-9	M [g/mol]	271.08
Structure	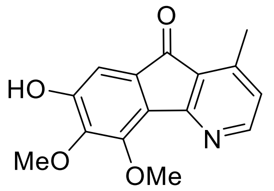	Chemical formula	$C_{15}H_{13}NO_4$
		Kováts index [iu]	2400
		RRT (fluorene)	1.546

Mass spectrum

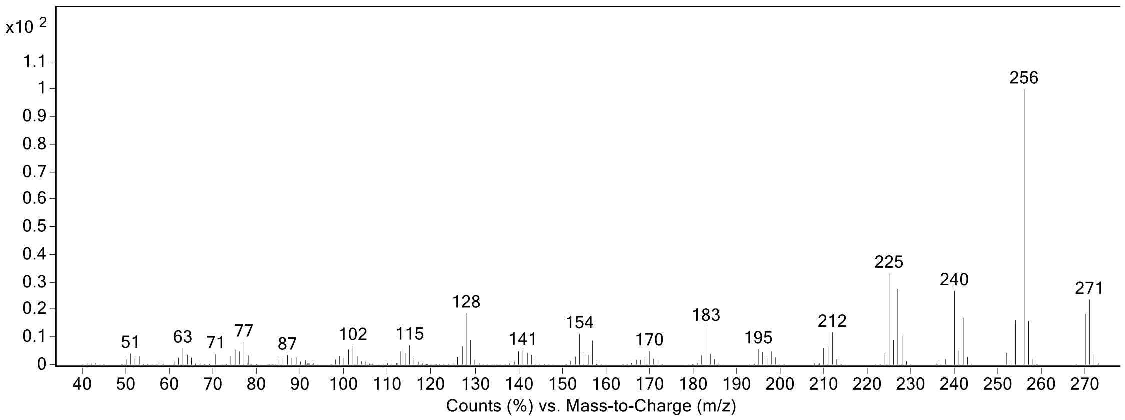

**Compound No.**	**15**		
**Trival name**	**Polyfothine**		
**CAS Number**	122908-91-2	**M [g/mol]**	255.09
**Structure**	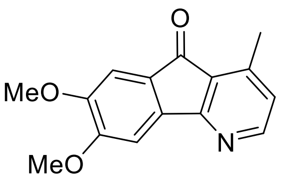	**Chemical formula**	$C_{15}H_{13}NO_3$
		**Kováts index [iu]**	2371
		**RRT (fluorene)**	1.529

Mass spectrum

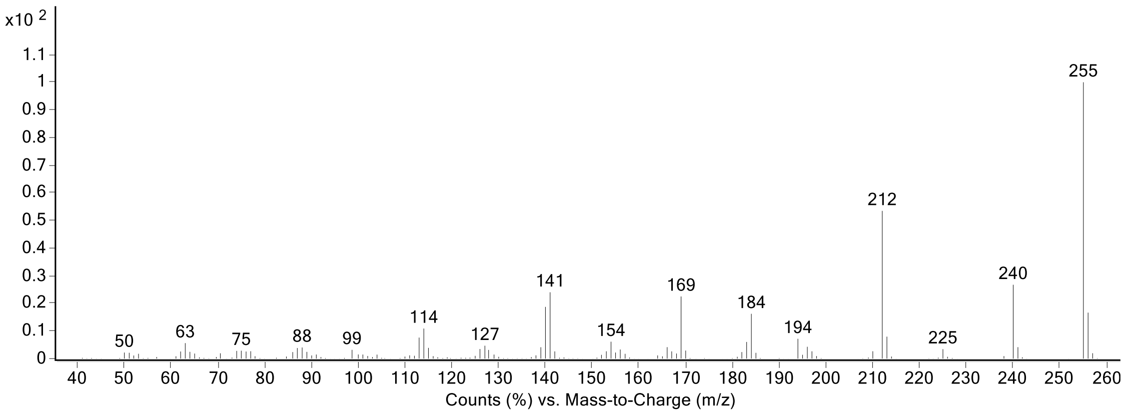

**Compound No.**	**16**		
**Trival name**	**5,6,7,8-Tetramethoxyonychine**		
**CAS Number**	-	**M [g/mol]**	315.11
**Structure**	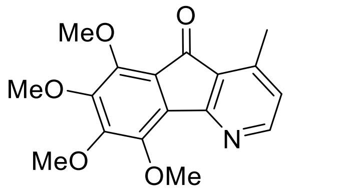	**Chemical formula**	$C_{17}H_{17}NO_5$
		**Kováts index [iu]**	2536
		**RRT (fluorene)**	1.621

Mass spectrum

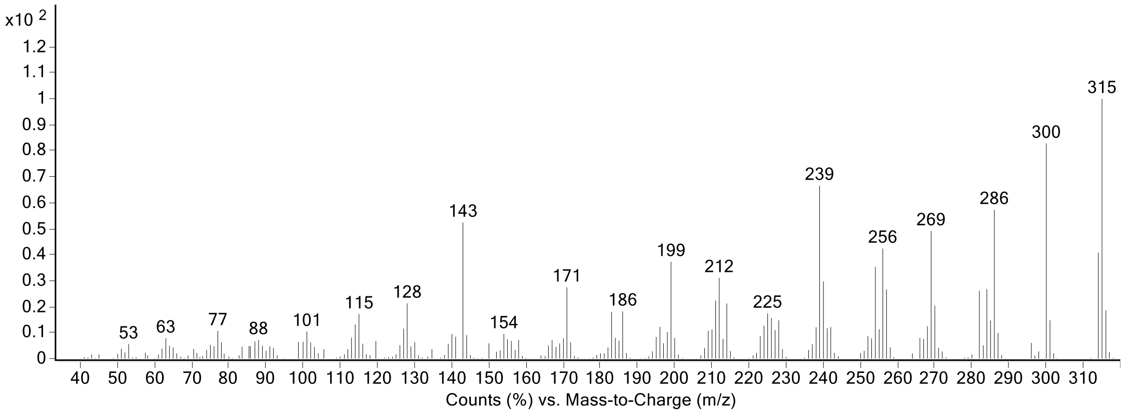

Compound No.			**17**
**Trival name**	**7-Hydroxy-5,8-dimethoxyonychine**		
**CAS Number**	-	**M [g/mol]**	271.08
**Structure**	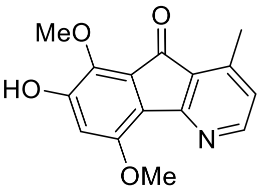	**Chemical formula**	$C_{15}H_{13}NO_4$
		**Kováts index [iu]**	2436
		**RRT (fluorene)**	1.566

Mass spectrum

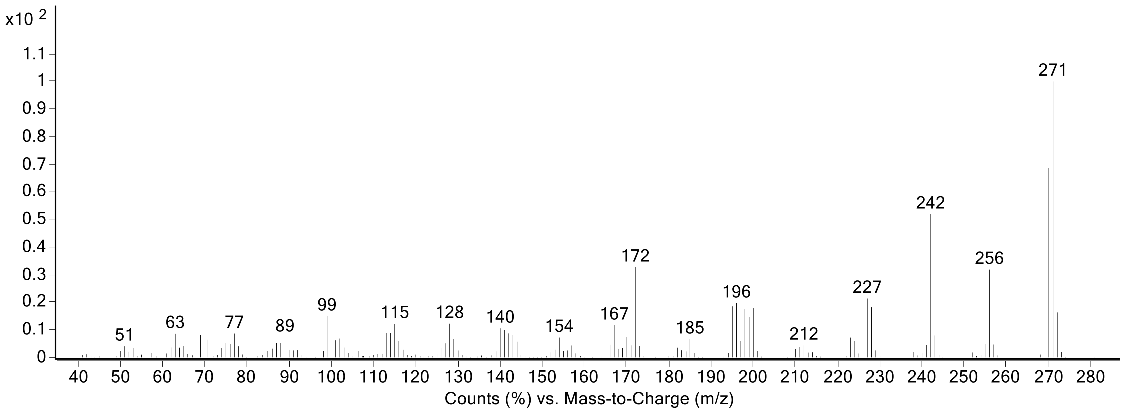

**Compound No.**	**18**		
**Trival name**	**7-Methoxyonychine**		
**CAS Number**	117719-70-7	**M [g/mol]**	225.08
**Structure**	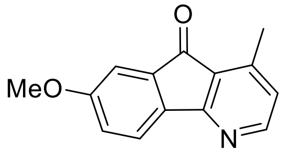	**Chemical formula**	$C_{14}H_{11}NO_2$
		**Kováts index [iu]**	2108
		**RRT (fluorene)**	1.369

Mass spectrum

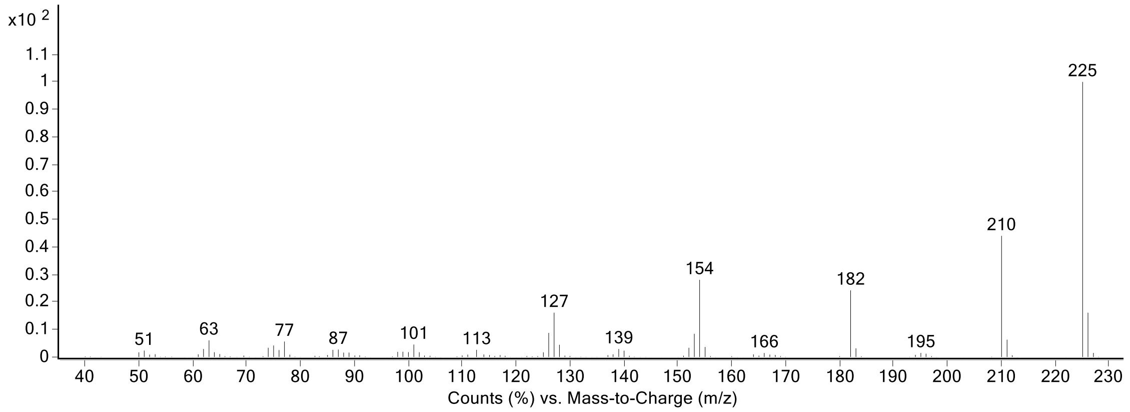

Compound No.	19		
Trival name	Muniranine		
CAS Number	-	M [g/mol]	301.10
Structure	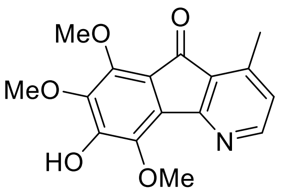	Chemical formula	$C_{16}H_{15}NO_5$
		Kováts index	2596
		RRT (fluorene)	1.653

Mass spectrum

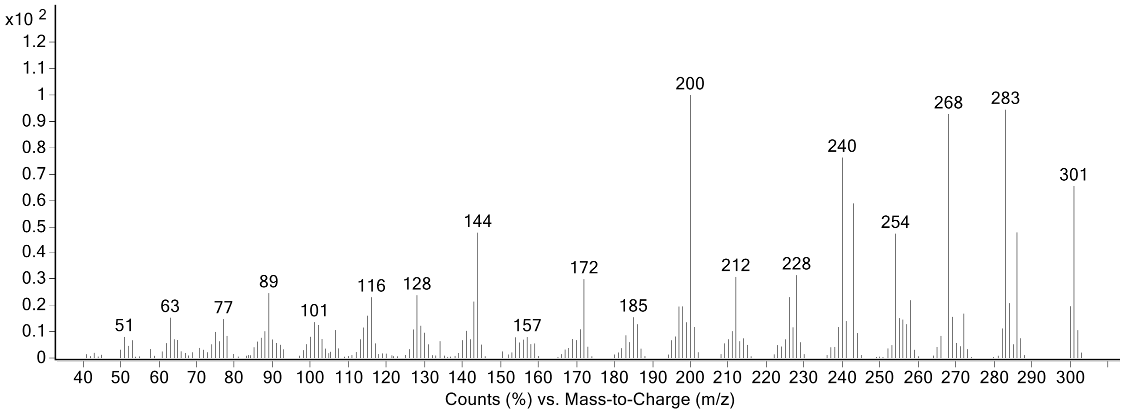

**Compound No.**	**20**		
**Trival name**	**5,6-Dimethoxyonychine**		
**CAS Number**	112368-58-8	**M [g/mol]**	255.09
**Structure**	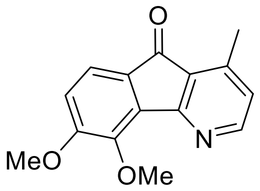	**Chemical formula**	$C_{15}H_{13}NO_3$
		**Kováts index [iu]**	2312
		**RRT (fluorene)**	1.494

Mass spectrum

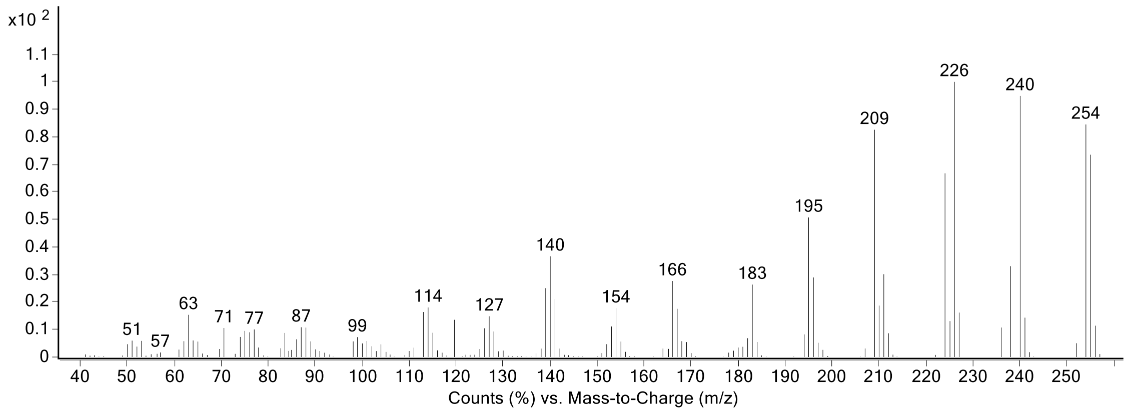

Compound No.			**21**
**Trival name**	**3-Methoxyonychine**		
**CAS Number**	145013-64-5	**M [g/mol]**	225.08
**Structure**	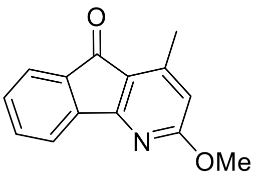	**Chemical formula**	$C_{14}H_{11}NO_2$
		**Kováts index [iu]**	1989
		**RRT (fluorene)**	1.290

Mass spectrum

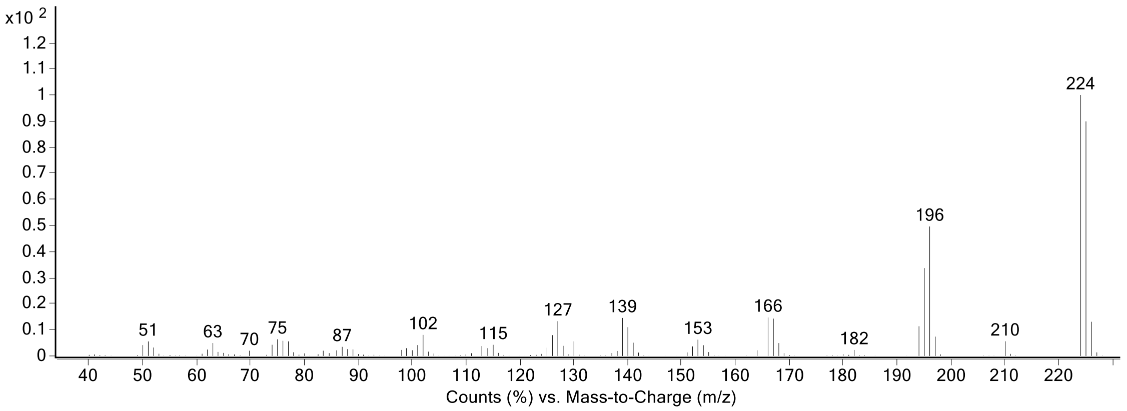

**Compound No.**			**22**
**Trival name**	**5,8-Dimethoxyonychine**		
**CAS Number**	112368-60-2	**M [g/mol]**	255.09
**Structure**	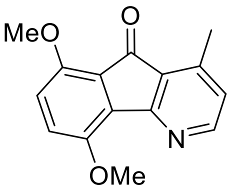	**Chemical formula**	$C_{15}H_{13}NO_3$
		**Kováts index [iu]**	2349
		**RRT (fluorene)**	1.516

Mass spectrum

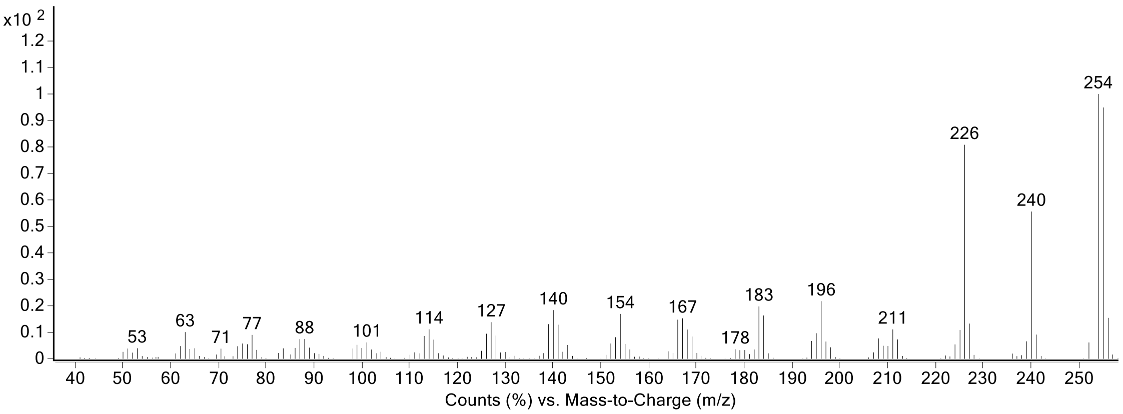

**Compound No.**	**23**		
**Trival name**	**5,7,8-Trimethoxyonychine**		
**CAS Number**	-	**M [g/mol]**	285.10
**Structure**	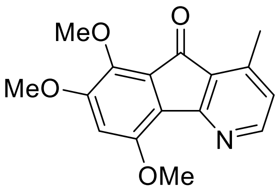	**Chemical formula**	$C_{16}H_{15}NO_4$
		**Kováts index [iu]**	2519
		**RRT (fluorene)**	1.612

Mass spectrum

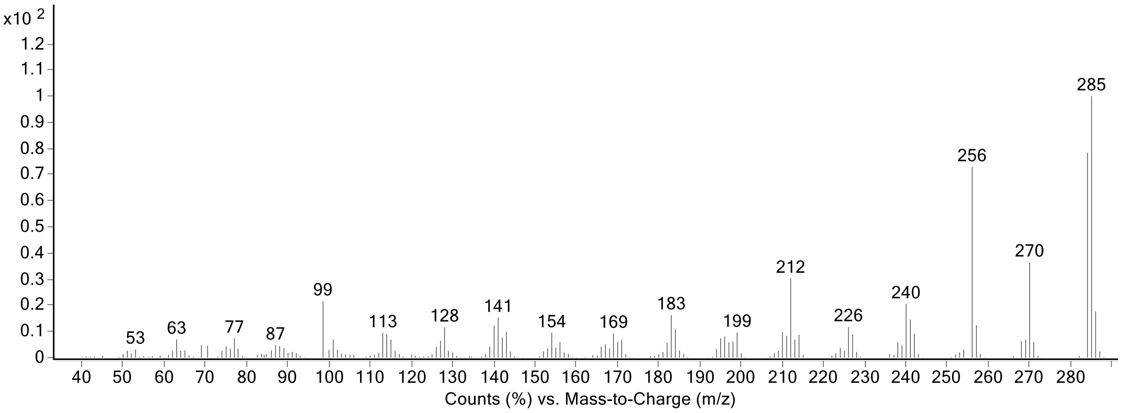

Compound No.	24		
Trival name	Polynemoraline C		
CAS Number	1129491-73-1	M [g/mol]	271.09
Structure	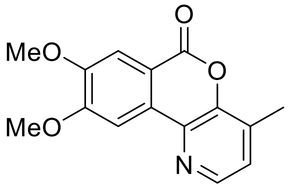	Chemical formula	$C_{15}H_{13}NO_4$
		Kováts index [iu]	2528
		RRT (fluorene)	1.617

Mass spectrum

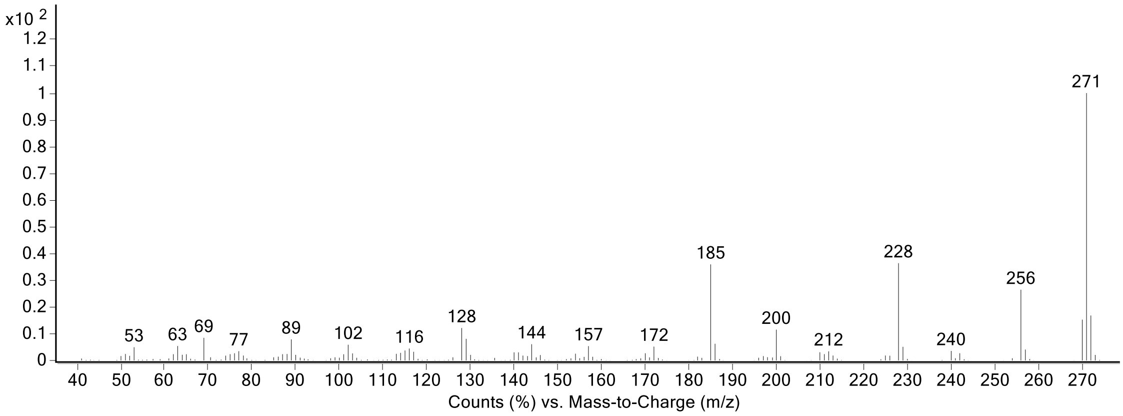

Compound No.	25		
Trival name	Annomontine		
CAS Number	82504-00-5	M [g/mol]	261.10
Structure	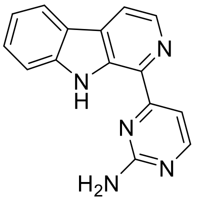	Chemical formula	$C_{15}H_{11}N_5$
		Kováts index [iu]	2920
		RRT (fluorene)	1.816

Mass spectrum

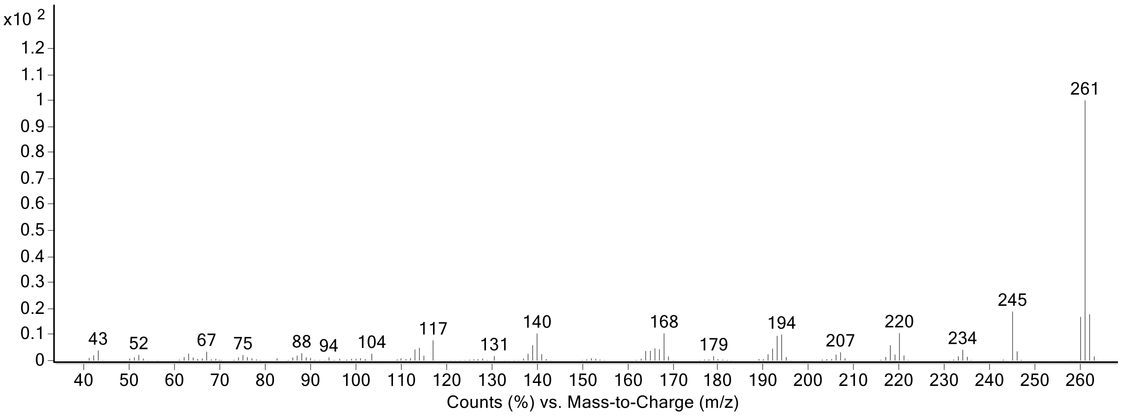

Supplement: Supplementary file 1 [file molecules-27-08217-s001.zip › Supplementary Material File S2. Substance data sheets.pdf]
