# Supplementary material for: A GC-MS Protocol for the Identification of Polycyclic Aromatic Alkaloids from Annonaceae
Source: Molecules. 2022 Nov 25;27(23):8217. doi: 10.3390/molecules27238217 (PMC9738936; doi:10.3390/molecules27238217)
Supplement: Supplementary file 1 [file molecules-27-08217-s001.zip › Table S1. Additional chromatographic parameters of the analytes.pdf]

Table S1: Additional chromatographic parameters of the analytes; * hold up time 1.259 min

Code	Trivial name		Characteristic ions [*m*/*z*] (relative intensities)	Retention time [min]	Retention factor (*k*)*	Separation factor (α)	Peak width at base [min]	Peak resolution (R_s)
**1**	Annocherine A	**(I)**	280 (100)<br>265 (11)<br>220 (7)	25.97	19.6	2.88	0.16	0.1
		**(II)**	279 (100)<br>264 (50)<br>236 (45)	28.34	21.5	2.58	0.19	1.7
**2**	Annocherine B	**(I)**	280 (100)<br>265 (10)<br>220 (10)	25.97	19.6	3.09	0.20	0.0
		**(II)**	279 (100)<br>264 (49)<br>236 (47)	28.35	21.5	4.26	0.27	0.0
**3**	*O*,*O*-Dimethylannocherine A	**(I)**	322 (38)<br>308 (74)<br>292 (100)	26.77	20.2	0.47	0.16	5.2
		**(II)**	308 (100)<br>294 (83)<br>278 (14)	24.98	18.9	0.94	0.15	6.9
		**(III)**	307 (92)<br>292 (100)<br>248 (26)	27.68	21.0	0.78	0.78	1.7
**4**	Lysicamine		291 (98)<br>248 (100)<br>177 (24)	28.08	21.3	2.74	0.16	1.2
**5**	Sampangine		232 (100)<br>204 (79)<br>151 (12)	23.76	17.9	0.69	0.13	0.8
**6**	Eupolauridine		204 (100)<br>177 (13)<br>150 (7)	19.28	14.3	0.28	0.15	5.9
**7**	Eupolauridine mono-*N*-oxide		220 (66)<br>204 (100)<br>165 (20)	23.35	17.5	0.70	0.18	1.2
**8**	Eupolauridine di-*N*-oxide		236 (48)<br>220 (22)<br>204 (100)	26.92	20.4	0.75	0.10	1.4
**9**	Cleistopholine		223 (100)<br>195 (75)<br>167 (36)	20.94	15.6	0.40	0.15	3.9
**10**	Onychine		195 (100)<br>166 (21)<br>139 (21)	16.73	12.3	0.79	0.17	1.1
**11**	Ursuline		241 (100)<br>223 (74)<br>183 (64)	20.41	15.2	0.56	0.12	5.7
**12**	Isoursuline		241 (99)<br>212 (100)<br>198 (71)	21.16	15.8	0.60	0.15	1.4

Code	Trivial name	Characteristic ions [*m*/*z*] (relative intensities)	Retention time [min]	Retention factor (*k*)	Separation factor (α)	Peak width at base [min]	Peak resolution (R_s)
**13**	6-Methoxyonychine	225 (100)<br>182 (15)<br>154 (15)	19.77	14.7	0.50	0.11	1.9
**14**	Darienine	271 (23)<br>256 (100)<br>225 (33)	22.12	16.5	0.41	0.15	2.1
**15**	Polyfothine	255 (100)<br>212 (53)<br>169 (21)	21.83	16.3	0.51	0.13	1.0
**16**	5,6,7,8-Tetramethoxyonychine	315 (100)<br>300 (87)<br>239(68)	23.15	17.4	0.20	0.16	1.0
**17**	7-Hydroxy-5,8-dimethoxyonychine	271 (100)<br>242 (51)<br>172 (37)	22.41	16.8	0.36	0.17	1.1
**18**	7-Methoxyonychine	255 (100)<br>210 (42)<br>154 (28)	19.57	14.5	0.34	0.11	2.3
**19**	Muniranine	301 (68)<br>283 (94)<br>200 (100)	23.65	17.8	0.46	0.12	2.0
**20**	5,6-Dimethoxyonychine	255 (71)<br>254 (83)<br>226 (100)	21.37	15.9	0.49	0.13	0.9
**21**	3-Methoxyonychine	225 (91)<br>224 (100)<br>196 (51)	18.44	13.6	0.46	0.13	9.0
**22**	5,8-Dimethoxyonychine	255 (92)<br>254 (100)<br>226 (84)	21.69	16.2	0.40	0.16	2.2
**23**	5,7,8-Trimethoxyonychine	285 (100)<br>270 (34)<br>256 (67)	23.02	17.3	0.60	0.11	4.3
**24**	Polynemoraline C	271 (100)<br>228 (40)<br>185 (38)	23.15	17.3	0.33	0.17	0.9
**25**	Annomontine	261 (100)<br>245 (19)<br>220 (11)	25.96	19.6	0.48	0.15	1.7
