# Supplementary figures and images for: A GC-MS Protocol for the Identification of Polycyclic Aromatic Alkaloids from Annonaceae
Source: Molecules. 2022 Nov 25;27(23):8217. doi: 10.3390/molecules27238217 (PMC9738936; doi:10.3390/molecules27238217)

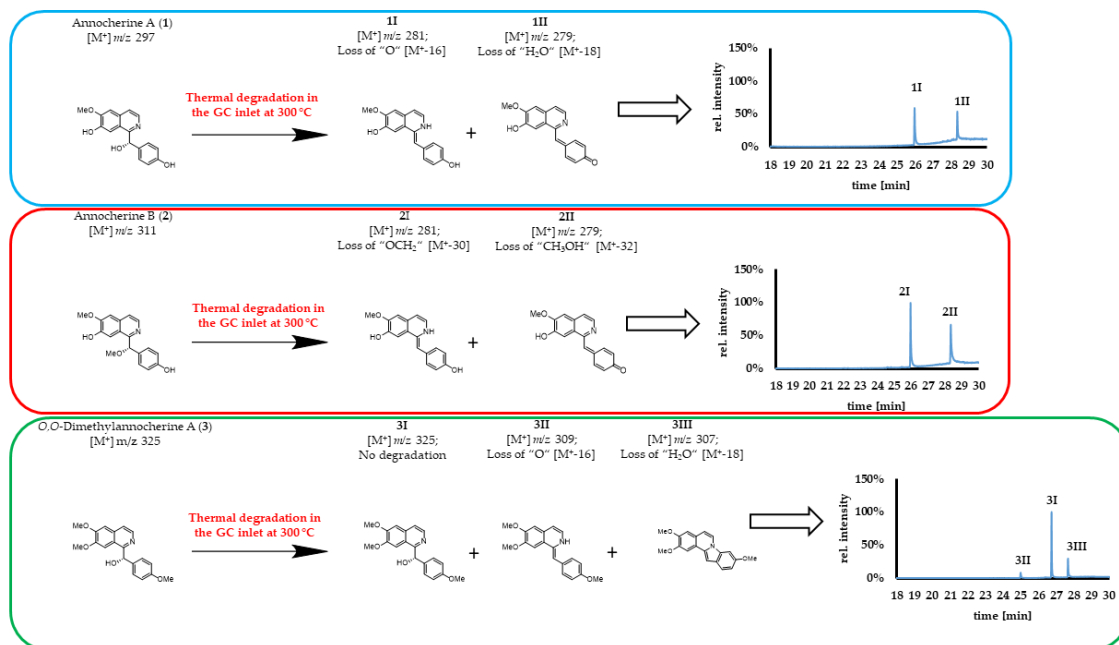

Figure S1: Degradation; Table S1: Additional chromatographic parameters of the analytes.

Supplement: Supplementary file 1 [file molecules-27-08217-s001.zip › molecules-2030983-supplementary.pdf]
